# Wikipedia: Why is the common knowledge resource still neglected by academics?

**DOI:** 10.1093/gigascience/giz139

**Published:** 2019-12-03

**Authors:** Dariusz Jemielniak

**Affiliations:** Kozminski University, Management in Networked and Digital Societies (MINDS) department, Jagiellonska 59, 03301 Warszawa, Poland

**Keywords:** Wikipedia, academia, online encyclopedia, knowledge quality, free knowledge

## Abstract

Wikipedia is by far the largest online encyclopedia, and the number of errors it contains is on par with the professional sources even in specialized topics such as biology or medicine. Yet, the academic world is still treating it with great skepticism because of the types of inaccuracies present there, the widespread plagiarism from Wikipedia, and historic biases, as well as jealousy regarding the loss of the knowledge dissemination monopoly. This article argues that it is high time not only to acknowledge Wikipedia's quality but also to start actively promoting its use and development in academia.

## Background

In 2005, *Nature* published a study describing Wikipedia as going “head to head” with Britannica [1]. While the claim was disputed by Britannica, since then Wikipedia has grown 6-fold in the number of articles; is >85 times the size of 120-volume *Encyclopedia Britannica*, measured by word count; and has substantially improved its quality.

Admittedly, standards of quality are shaped by peer-to-peer local language communities and vary widely among Wikipedia projects, and also between articles within languages [2]. Yet, the quality of Wikipedia articles is very high [3]. This is true even in many specialized topics, such as anatomy, biology, or medicine, where Wikipedia is as accurate as the professional sources [4–6], even though sometimes it does not score high on readability.

Yet, Wikipedia is still treated with suspicion by the professoriate and sneered at in academic circles [7]. This is especially disturbing, as academics are best positioned to shape Wikipedia [8], because of their expertise, as well as because of their access to students, who can improve Wikipedia for coursework under their supervision. Thus, it may be worthwhile to consider the reasons for scholars’ reluctance to openly use, recommend, and incorporate Wikipedia into coursework.

## Main Text

Some of the reasons for these reservations may be legitimate. Although Wikipedia has a similar number of errors to professional and peer-reviewed sources [4–6], the types of inaccuracies on Wikipedia are different. They may involve replacing the content of an article with nonsense, or someone's name with a slur. There is no question that such vandalism damages the perception of the quality of Wikipedia as a whole. Still, Wikipedia takes vandalism seriously and constantly develops new methods of combating malicious edits, including, e.g., machine learning algorithms, as well as human patrolling. The sorts of vandalism that pass through may misinform the readers but are overall quite rare, especially in popular articles. More importantly, most vandalism is easily spotted and as such is harmful mainly to the image of Wikipedia as a trustworthy source, and does not actually misinform the readers.

Another reason for academia's dislike of Wikipedia may be its association with plagiarism. Students are notorious for copying from Wikipedia. However, this is clearly an unfortunate testimony to its quality and should not be held against Wikipedia, just as it should not be held against any other plagiarized academic resource. On a side note, Wikipedia has iron-clad copyright policies and treats plagiarism more seriously than regular media.

Some other reasons may be related to a historic bias, a perception of Wikipedia as not rigorous enough, or underestimation of the ability of amateurs to disseminate knowledge in a robust way. As scholars, we should be able to confront and eliminate such biases once we are presented with evidence, and many studies show that Wikipedia delivers high-quality output in practice, even if in theory it may seem impossible. Wikipedia simply is a living testament to Linus's Law: “given enough eyeballs, all bugs are shallow,” and the more edited articles are actually more accurate. It may be surprising and strange, but the results speak for themselves. Over time Wikipedia's quality has improved substantially, and yet it is still perceived in a static and dated way, as from the time of its inception.

Some professors dislike it when students cite Wikipedia. While no encyclopedia should be the only source in academic-level essays, it should be emphasized that our primary duty is to report and accurately refer to all sources that were actually used, with no exceptions. Academic honesty and transparency are crucial for scholarly work, and it is difficult to understand why citing specifically Wikipedia is taboo.

Yet, the most important reason for animosity towards Wikipedia may be that it challenges the existing institutional hierarchy of knowledge distribution and is much more successful in reaching the public than academic publications. We, the professors, were the only ones legitimized to disseminate academic knowledge. Now, we have to compete with a product of anonymous amateurs, which has a readership much wider than any of us could ever dream of. In fact, Wikipedia systematically compensates for the lack of credentials by heavy emphasis on reliable sources. It is a paradox: Wikipedia is one of the 10 most popular websites in the world according to TopSites, and by most measures it is the most widely read knowledge repository on Earth, but still it is often treated as not worth academic attention.

We need to change this. Writing a Wikipedia article is a perfect academic assignment for students. It requires finding reliable, verifiable sources, synthesizing their content, writing an encyclopedic entry: a true paragon of scholarly effort and transferable information literacy skills. Moreover, it makes the professor's life so much easier because a new article is often checked for plagiarism and commented on by members of the community. However, I believe there are even more important reasons for students and scholars to appreciate Wikipedia. Billions of people do not have access to free knowledge. We are the 1% in terms of knowledge access privilege; developing Wikipedia, the common good of humanity, is our moral obligation. The fact that Wikipedia development makes our coursework easier is only a nice bonus.

## Conclusions

There are already initiatives in computational biology or genetics aimed at developing Wikipedia articles from these topics by scholars [9]. GeneWiki project, established to transfer information about relationships and functions of all human genes from scientific resources to Wikipedia, already contains 10,000 distinct gene pages, viewed >50 million times per year [10]. Nevertheless, Wikipedia development is not yet routinely considered as valuable in tenure reviews, and Wikipedia article writing is not yet a mainstream coursework assignment in colleges. It is high time to make that happen. In 2019 Wikipedia turned 18, so maybe academics should start treating it as an adult.

## Competing interests

The author is a member of the Wikimedia Foundation Board of Trustees.

## Author's information

D.J. is Professor and Head of the Management in Networked and Digital Societies (MINDS) department at Kozminski University, associate faculty at Berkman-Klein Center for Internet and Society at Harvard University, and fellow at MIT Center for Collective Intelligence. He serves on the Wikimedia Foundation Board of Trustees. In 2014 he published *Common Knowledge? An Ethnography of Wikipedia* (Stanford University Press).

## Funding

Working on this article was possible thanks to grant No. PPN/BEK/2018/1/00009 from the Polish National Agency for Academic Exchange.

## Supplementary Material

giz139_GIGA-D-19-00332_Original_Submission

giz139_GIGA-D-19-00332_Revision_1

giz139_Response_to_Reviewer_Comments_Original_Submission

giz139_Reviewer_1_Report_Original_Submission Thomas Shafee -- 10/18/2019 Reviewed

giz139_Supplemental_Files
