# Supplementary material for: Wikipedia: Why is the common knowledge resource still neglected by academics?
Source: Gigascience. 2019 Dec 3;8(12):giz139. doi: 10.1093/gigascience/giz139 (PMC6889752; doi:10.1093/gigascience/giz139)
Supplement: giz139_GIGA-D-19-00332_Original_Submission [file giz139_giga-d-19-00332_original_submission.pdf]

GigaScience

Wikipedia: why is the common knowledge resource still neglected by academics?

--Manuscript Draft--

**Manuscript Number:**	GIGA-D-19-00332
**Full Title:**	Wikipedia: why is the common knowledge resource still neglected by academics?
**Article Type:**	Commentary
**Funding Information:**	Polish National Agency for Academic Exchange (PPN/BEK/2018/1/00009) — Prof. Dr Dariusz Jemielniak
**Abstract:**	Wikipedia is by far the largest online encyclopedia, and the number of errors it contains is on par with the professional sources even in specialized topics such as biology or medicine. Yet, the academic world is still treating it with great skepticism, because of the types of inaccuracies present there, the widespread plagiarism from Wikipedia, historic biases, as well as jealousy for the loss of knowledge dissemination monopoly. This article argues that it is high time not only to acknowledge Wikipedia's quality but also start actively promoting its use and development in academia.
**Corresponding Author:**	Dariusz Jemielniak<br>Massachusetts Institute of Technology<br>Cambridge, MA UNITED STATES
**Corresponding Author Secondary Information:**	
**Corresponding Author's Institution:**	Massachusetts Institute of Technology
**Corresponding Author's Secondary Institution:**	
**First Author:**	Dariusz Jemielniak
**First Author Secondary Information:**	
**Order of Authors:**	Dariusz Jemielniak
**Order of Authors Secondary Information:**	
**Additional Information:**	
**Question**	**Response**
Are you submitting this manuscript to a special series or article collection?	No
**Experimental design and statistics**<br><br>Full details of the experimental design and statistical methods used should be given in the Methods section, as detailed in our Minimum Standards Reporting Checklist. Information essential to interpreting the data presented should be made available in the figure legends.<br><br>Have you included all the information requested in your manuscript?	Yes

Powered by Editorial Manager® and ProduXion Manager® from Aries Systems Corporation

**Resources**<br><br>A description of all resources used, including antibodies, cell lines, animals and software tools, with enough information to allow them to be uniquely identified, should be included in the Methods section. Authors are strongly encouraged to cite Research Resource Identifiers (RRIDs) for antibodies, model organisms and tools, where possible.<br><br>Have you included the information requested as detailed in our Minimum Standards Reporting Checklist?	Yes
**Availability of data and materials**<br><br>All datasets and code on which the conclusions of the paper rely must be either included in your submission or deposited in publicly available repositories (where available and ethically appropriate), referencing such data using a unique identifier in the references and in the “Availability of Data and Materials” section of your manuscript.<br><br>Have you have met the above requirement as detailed in our Minimum Standards Reporting Checklist?	Yes

Powered by Editorial Manager® and ProduXion Manager® from Aries Systems Corporation

Click here to download Manuscript GigaScience -Wikipedia edited.docx

Wikipedia: why is the common knowledge resource still neglected by academics?

Dariusz Jemielniak

Center for Collective Intelligence, Massachusetts Institute of Technology

dariuszj@mit.edu

Abstract

Wikipedia is by far the largest online encyclopedia, and the number of errors it contains is on par with the professional sources even in specialized topics such as biology or medicine. Yet, the academic world is still treating it with great skepticism, because of the types of inaccuracies present there, the widespread plagiarism from Wikipedia, historic biases, as well as jealousy for the loss of knowledge dissemination monopoly. This article argues that it is high time not only to acknowledge Wikipedia's quality but also start actively promoting its use and development in academia.

Keywords

Wikipedia, Academia, online encyclopedia, knowledge quality, free knowledge

Background

In 2005, *Nature* published a study describing Wikipedia as going "head to head" with Britannica [1]. While the claim was disputed by Britannica, since then Wikipedia has grown sixfold in the number of articles and is more than 85 times the size of 120-volume *Encyclopedia Britannica,* measured by word count, and has significantly improved its quality.

Admittedly, standards of quality are shaped by peer-to-peer local language communities and vary largely among Wikipedia projects [2]. Yet, the quality of Wikipedia articles is very high [3]. This is true even in very specialized topics, such as anatomy, biology or medicine where Wikipedia is as accurate as the professional sources [4–6], even though sometimes it does not score high on readability.

Yet, Wikipedia is still treated with suspicion by the professoriate and sneered at in academic circles [7]. This is especially disturbing, as academics are best positioned to shape Wikipedia [8], because of their expertise, as well as because of their access to students, who can improve Wikipedia for classwork under their supervision. Thus, It may be worthwhile to consider the reasons for the scholars' reluctance to openly use, recommend, and incorporate Wikipedia into coursework.

Main text

Some of the reasons for these reservations may be legitimate. Although Wikipedia has a similar number of errors as professional and peer-reviewed sources [4–6], the types of inaccuracies on Wikipedia are different. They may involve replacing the content of an article with nonsense, or someone's name with a slur. There is no question that such vandalism damages the perception of the quality of Wikipedia as a whole. Still, most vandalism in popular articles is removed in a matter of seconds. In addition, Wikipedia takes vandalism seriously and constantly develops new methods of combating malicious edits, including for instance machine learning algorithms. More importantly, most vandalism is easily spotted and as such

is harmful mainly to the image of Wikipedia as a trustworthy source, and does not actually misinform the readers.

Other reasons for academia's dislike of Wikipedia may be its association with plagiarism. Students are notorious for copying from Wikipedia. However, it is quite clearly an unfortunate testimony to its quality and should not be held against Wikipedia, just as it should not be held against any other plagiarized academic resource. On a side note, Wikipedia has iron-clad copyright policies and treats plagiarism more seriously than regular media.

Some other reasons may be related to a historic bias, a perception of Wikipedia as not rigorous enough, or underestimation of the ability of amateurs to disseminate knowledge in a robust way. As scholars, we should be able to confront and eliminate such biases once we are presented with evidence, and many studies show that Wikipedia delivers high-quality output in practice, even if in theory it may seem impossible. Wikipedia simply is a living testament to Linus's Law: “given enough eyeballs, all bugs are shallow". It may be surprising and strange, but the results speak for themselves.

Some professors dislike it when students cite Wikipedia. While no encyclopedia should be the only source in academic-level essays, it should be emphasized that our primary duty is to report and accurately refer to all sources that were actually used, with no exceptions.

Yet, the most important reason for animosity towards Wikipedia may be that it challenges the existing institutional hierarchy of knowledge distribution, and is much more successful in reaching the public than academic publications. We, the professors, were the only ones legitimized to disseminate academic knowledge. Now, we have to compete with a product of anonymous amateurs, which has a readership much wider than any of us could ever dream of. It is a paradox: Wikipedia is one of the ten most popular websites in the world according to TopSites, and by most measures it is the most widely read knowledge repository on Earth, and still it is often treated as not worth academic attention.

We need to change this. Writing a Wikipedia article is a perfect academic assignment for students. It requires finding reliable, verifiable sources, synthesizing their content, writing an encyclopedic entry: a true paragon of scholarly effort. Moreover, it makes the professor's life so much easier, as a new article is often checked for plagiarism and commented on by members of the community. However, I believe there are even more important reasons for students and scholars to appreciate Wikipedia. Billions of people do not have access to free knowledge. We are the 1% in terms of knowledge access privilege; developing Wikipedia, the common good of humanity, is our moral obligation. The fact that Wikipedia development makes our coursework easier is only a nice bonus.

Conclusions

There are already initiatives in computational biology or genetics aimed at developing Wikipedia articles from these topics by scholars [9]. GeneWiki project, established to transfer information about relationships and functions of all human genes from scientific resources to Wikipedia already contains 10,000 distinct gene pages, viewed over 50 million times per year [10]. Nevertheless, Wikipedia development is not yet routinely considered as valuable in tenure reviews, and Wikipedia article writing is not yet a mainstream coursework assignment in colleges. It is high time to make that happen. In 2019 Wikipedia turned 18, so maybe academics should start treating it as an adult.

Declarations

Ethics approval and consent to participate: not applicable

Consent for publication: not applicable

Availability of data and material: not applicable

Competing interests: The author is a member of the Wikimedia Foundation Board of Trustees

Funding: Working on this article was possible thanks to a grant no. PPN/BEK/2018/1/00009 from Polish National Agency for Academic Exchange.

Authors' contributions: not applicable

Acknowledgments: not applicable

Author's information: DJ is Professor and Head of Management in Networked and Digital Societies (MINDS) department at Kozminski University, associate faculty at Berkman-Klein Center for Internet and Society at Harvard University, and fellow at MIT Center for Collective Intelligence. He serves on the Wikimedia Foundation Board of Trustees. In 2014 he published *Common Knowledge? An Ethnography of Wikipedia* (Stanford University Press).

References

1. Giles J. Internet encyclopaedias go head to head. Nature. 2005;438:900–1.

2. Jemielniak D, Wilamowski M. Cultural diversity of quality of information on Wikipedias. Journal of the Association for Information Science and Technology. 2017;68:2460–70.

3. Michelucci P, Dickinson JL. The power of crowds. Science. 2016;351:32–3.

4. James R. WikiProject Medicine: Creating Credibility in Consumer Health. J Hosp Librariansh. 2016;16:344–51.

5. Mesgari M, Okoli C, Mehdi M, Nielsen FÅ, Lanamäki A. "The sum of all human knowledge": A systematic review of scholarly research on the content of Wikipedia. Journal of the Association for Information Science and Technology. 2015;66:219–45.

6. London DA, Andelman SM, Christiano AV, Kim JH, Hausman MR, Kim JM. Is Wikipedia a complete and accurate source for musculoskeletal anatomy? Surg Radiol Anat [Internet]. 2019; Available from: http://dx.doi.org/10.1007/s00276-019-02280-1

7. Jemielniak D, Aibar E. Bridging the gap between Wikipedia and academia. Journal of the Association for Information Science and Technology. 2016;67:1773–6.

8. Shafee T, Mietchen D, Su AI. Academics can help shape Wikipedia. Science. 2017;357:557–8.

9. Mietchen D, Wodak S, Wasik S, Szostak N, Dessimoz C. Submit a Topic Page to PLOS Computational Biology and Wikipedia. PLoS Comput Biol. 2018;14:e1006137.

10. Tsueng G, Good BM, Ping P, Golemis E, Hanukoglu I, van Wijnen AJ, et al. Gene Wiki Reviews-Raising the quality and accessibility of information about the human genome. Gene. 2016;592:235–8.
