# Supplementary material for: Wikipedia: Why is the common knowledge resource still neglected by academics?
Source: Gigascience. 2019 Dec 3;8(12):giz139. doi: 10.1093/gigascience/giz139 (PMC6889752; doi:10.1093/gigascience/giz139)
Supplement: giz139_GIGA-D-19-00332_Revision_1 [file giz139_giga-d-19-00332_revision_1.pdf]

GigaScience

Wikipedia: why is the common knowledge resource still neglected by academics?

--Manuscript Draft--

**Manuscript Number:**	GIGA-D-19-00332R1
**Full Title:**	Wikipedia: why is the common knowledge resource still neglected by academics?
**Article Type:**	Commentary
**Funding Information:**	Polish National Agency for Academic Exchange (PPN/BEK/2018/1/00009) — Not applicable
**Abstract:**	Wikipedia is by far the largest online encyclopedia, and the number of errors it contains is on par with the professional sources even in specialized topics such as biology or medicine. Yet, the academic world is still treating it with great skepticism, because of the types of inaccuracies present there, the widespread plagiarism from Wikipedia, historic biases, as well as jealousy for the loss of knowledge dissemination monopoly. This article argues that it is high time not only to acknowledge Wikipedia's quality but also start actively promoting its use and development in academia.
**Corresponding Author:**	Dariusz Jemielniak<br>Akademia Leona Kozminskiego<br>Warsaw, POLAND
**Corresponding Author Secondary Information:**	
**Corresponding Author's Institution:**	Akademia Leona Kozminskiego
**Corresponding Author's Secondary Institution:**	
**First Author:**	Dariusz Jemielniak
**First Author Secondary Information:**	
**Order of Authors:**	Dariusz Jemielniak
**Order of Authors Secondary Information:**	
**Response to Reviewers:**	I appreciate the Reviewer's comments and I'm implementing the suggested changes in full. Comments in-line (as well as in a separate file, for reading clarity):<br><br>> Reviewer #1: This is a clearly written and timely commentary. It posits that academic engagement in Wikipedia is below where it should be, and speculates on the main reasons why (with the author's opinions on appropriate rebuttals). The author has been appropriate in using recent references (since Wikipedia changes relatively rapidly) and older references are interpreted in context.<br><br>My only major comment is that the issue is still presented as static. Has Wikipedia's quality clanged over time? Have the reasons for its neglect also changed, or remained static? It may be worth separating in the "historic bias" paragraph which points are relevant to bias from long-term historical perceptions on what constitutes authority, or from shorter-term perceptions based on Wikipedia's early reputation.<br><br>-----------<br>For space limitations, I'm reluctant to write a whole paragraph, but to address this issue I'm adding a sentence:<br>Over time Wikipedia's quality improved significantly, and yet it is still perceived in a static way, as from the times of its inception.<br>-----------<br><br>Background<br><br>P2: It may be worth noting that there is considerable variation in quality between articles within languages.

Good point. Adding "…, and also between articles within languages" to the sentence: Admittedly, standards of quality are shaped by peer-to-peer local language communities and vary largely among Wikipedia projects, and also between articles within languages
----

P2: "even in very specialized topics" should eb qualified as either in "many specialized topics" or "often in". there have been sources that have found against Wikipedia's quality in some cases. It is also worth noting that the references supporting accuracy in specialised topics often focus on more highly-trafficked pages. Nevertheless, the majority of Wikipedia articles remain 'stub' or 'start' quality level.

Agreed, changed to "many specialized topics" per suggestion.

Main text
P1: The authors make a good point that the 'anyone can edit' nature can cause suspicion in academics. Is there any known correlation between more-edited articles and accuracy?

This is an excellent question that does not have, up to my knowledge, a simple answer, as accuracy is measured differently across topics. Quite a few authors claim that editing leads to rapid improvement, and according to Clay Shirky stubs are much more likely to foster article development than red links. Given that we're at capacity with references, I'm reluctant to discuss this issue in the article, as we do not have solid universal number to support it.

P1: The argument that vandalism that is not immediately picked up by bots is mainly obvious and doesn't misinform readers is a risky. I think it would be more appropriate to reverse the sentence and note the machine learning and human editor patrols first (and hence the fast reversion rates), then concede that the sorts of vandalism that passes through may misinform readers but is orders of magniturde rarer.

Agreed, revised.

P3: It may be worth noting that the normal reliance on author credentials/reputation for authority is compensated for by the deliberate mechanism of heavy emphasis on reliable sources and verifiability as Wikipedia's foundation of authority.

Agreed, added: "In fact, Wikipedia systematically compensates for the lack of credentials by heavy emphasis on reliable sources."

P3: It may also be worth noting that despite the 'anyone can edit' fear, more-edited articles are actually tyically more accurate (Per Wilkinson and Huberman 2007. "Cooperation and quality in wikipedia").

Agreed, added for clarity to the sentence about Linus' law (…", and the more edited articles are actually more accurate").

P4: May be worth emphasising academic honest and transparency. Perhaps the taboo should be more accurately be against citing _only_ wikipedia.

	-----------<br>Agreed, added “Academic honesty and transparency are crucial for scholarly work, and it is difficult to understand why citing specifically Wikipedia is such a taboo.”<br>-----------<br><br>P6: Not only "paragon of scholarly effort" but transferable information literacy skills.<br>-----------<br>Agreed, added.<br>-----------
**Additional Information:**	
**Question**	**Response**
Are you submitting this manuscript to a special series or article collection?	No
**Experimental design and statistics**<br><br>Full details of the experimental design and statistical methods used should be given in the Methods section, as detailed in our Minimum Standards Reporting Checklist. Information essential to interpreting the data presented should be made available in the figure legends.<br><br>Have you included all the information requested in your manuscript?	Yes
**Resources**<br><br>A description of all resources used, including antibodies, cell lines, animals and software tools, with enough information to allow them to be uniquely identified, should be included in the Methods section. Authors are strongly encouraged to cite Research Resource Identifiers (RRIDs) for antibodies, model organisms and tools, where possible.<br><br>Have you included the information requested as detailed in our Minimum Standards Reporting Checklist?	Yes
**Availability of data and materials**<br><br>All datasets and code on which the conclusions of the paper rely must be	Yes

Powered by Editorial Manager® and ProduXion Manager® from Aries Systems Corporation

either included in your submission or deposited in publicly available repositories (where available and ethically appropriate), referencing such data using a unique identifier in the references and in the "Availability of Data and Materials" section of your manuscript.<br><br>Have you have met the above requirement as detailed in our Minimum Standards Reporting Checklist?	

Powered by Editorial Manager® and ProduXion Manager® from Aries Systems Corporation

Manuscript clean version

Click here to view linked References

Click here to access/download;Manuscript;GigaScience - Wikipedia_CLEAN.docx

Wikipedia: why is the common knowledge resource still neglected by academics?

Dariusz Jemielniak

Kozminski University, Management in Networked and Digital Societies (MINDS) department

darekj@kozminski.edu.pl

Abstract

Wikipedia is by far the largest online encyclopedia, and the number of errors it contains is on par with the professional sources even in specialized topics such as biology or medicine. Yet, the academic world is still treating it with great skepticism, because of the types of inaccuracies present there, the widespread plagiarism from Wikipedia, historic biases, as well as jealousy for the loss of knowledge dissemination monopoly. This article argues that it is high time not only to acknowledge Wikipedia's quality but also start actively promoting its use and development in academia.

Keywords

Wikipedia, Academia, online encyclopedia, knowledge quality, free knowledge

Background

In 2005, *Nature* published a study describing Wikipedia as going "head to head" with Britannica [1]. While the claim was disputed by Britannica, since then Wikipedia has grown sixfold in the number of articles and is more than 85 times the size of 120-volume *Encyclopedia Britannica,* measured by word count, and has significantly improved its quality.

Admittedly, standards of quality are shaped by peer-to-peer local language communities and vary largely among Wikipedia projects, and also between articles within languages [2]. Yet, the quality of Wikipedia articles is very high [3]. This is true even in many specialized topics, such as anatomy, biology or medicine where Wikipedia is as accurate as the professional sources [4–6], even though sometimes it does not score high on readability.

Yet, Wikipedia is still treated with suspicion by the professoriate and sneered at in academic circles [7]. This is especially disturbing, as academics are best positioned to shape Wikipedia [8], because of their expertise, as well as because of their access to students, who can improve Wikipedia for classwork under their supervision. Thus, It may be worthwhile to consider the reasons for the scholars' reluctance to openly use, recommend, and incorporate Wikipedia into coursework.

Main text

Some of the reasons for these reservations may be legitimate. Although Wikipedia has a similar number of errors as professional and peer-reviewed sources [4–6], the types of inaccuracies on Wikipedia are different. They may involve replacing the content of an article with nonsense, or someone's name with a slur. There is no question that such vandalism damages the perception of the quality of Wikipedia as a whole. Still, Wikipedia takes vandalism seriously and constantly develops new methods of combating malicious edits, including for instance machine learning algorithms, as well as human patrolling. The sorts of vandalism that pass through may misinform the readers, but are overall quite rare, especially in popular

articles.More importantly, most vandalism is easily spotted and as such is harmful mainly to the image of Wikipedia as a trustworthy source, and does not actually misinform the readers.

Other reasons for academia's dislike of Wikipedia may be its association with plagiarism. Students are notorious for copying from Wikipedia. However, it is quite clearly an unfortunate testimony to its quality and should not be held against Wikipedia, just as it should not be held against any other plagiarized academic resource. On a side note, Wikipedia has iron-clad copyright policies and treats plagiarism more seriously than regular media.

Some other reasons may be related to a historic bias, a perception of Wikipedia as not rigorous enough, or underestimation of the ability of amateurs to disseminate knowledge in a robust way. As scholars, we should be able to confront and eliminate such biases once we are presented with evidence, and many studies show that Wikipedia delivers high-quality output in practice, even if in theory it may seem impossible. Wikipedia simply is a living testament to Linus's Law: "given enough eyeballs, all bugs are shallow", and the more edited articles are actually more accurate. It may be surprising and strange, but the results speak for themselves. Over time Wikipedia's quality improved significantly, and yet it is still perceived in a static and dated way, as from the times of its inception.

Some professors dislike it when students cite Wikipedia. While no encyclopedia should be the only source in academic-level essays, it should be emphasized that our primary duty is to report and accurately refer to all sources that were actually used, with no exceptions. Academic honesty and transparency are crucial for scholarly work, and it is difficult to understand why citing specifically Wikipedia is such a taboo.

Yet, the most important reason for animosity towards Wikipedia may be that it challenges the existing institutional hierarchy of knowledge distribution, and is much more successful in reaching the public than academic publications. We, the professors, were the only ones legitimized to disseminate academic knowledge. Now, we have to compete with a product of anonymous amateurs, which has a readership much wider than any of us could ever dream of. In fact, Wikipedia systematically compensates for the lack of credentials by heavy emphasis on reliable sources. It is a paradox: Wikipedia is one of the ten most popular websites in the world according to TopSites, and by most measures it is the most widely read knowledge repository on Earth, and still it is often treated as not worth academic attention.

We need to change this. Writing a Wikipedia article is a perfect academic assignment for students. It requires finding reliable, verifiable sources, synthesizing their content, writing an encyclopedic entry: a true paragon of scholarly effort and transferable information literacy skills. Moreover, it makes the professor's life so much easier, as a new article is often checked for plagiarism and commented on by members of the community. However, I believe there are even more important reasons for students and scholars to appreciate Wikipedia. Billions of people do not have access to free knowledge. We are the 1% in terms of knowledge access privilege; developing Wikipedia, the common good of humanity, is our moral obligation. The fact that Wikipedia development makes our coursework easier is only a nice bonus.

Conclusions

There are already initiatives in computational biology or genetics aimed at developing Wikipedia articles from these topics by scholars [9]. GeneWiki project, established to transfer information about relationships and functions of all human genes from scientific resources to Wikipedia already contains 10,000 distinct gene pages, viewed over 50 million times per year [10]. Nevertheless, Wikipedia development is not yet routinely considered as valuable in tenure reviews, and Wikipedia article writing is not yet a mainstream coursework assignment in colleges. It is high time to make that happen. In 2019 Wikipedia turned 18, so maybe academics should start treating it as an adult.

Declarations

Ethics approval and consent to participate: not applicable

Consent for publication: not applicable

Availability of data and material: not applicable

Competing interests: The author is a member of the Wikimedia Foundation Board of Trustees

Funding: Working on this article was possible thanks to a grant no. PPN/BEK/2018/1/00009 from Polish National Agency for Academic Exchange.

Authors' contributions: not applicable

Acknowledgments: not applicable

Author’s information: DJ is Professor and Head of Management in Networked and Digital Societies (MINDS) department at Kozminski University, associate faculty at Berkman-Klein Center for Internet and Society at Harvard University, and fellow at MIT Center for Collective Intelligence. He serves on the Wikimedia Foundation Board of Trustees. In 2014 he published *Common Knowledge? An Ethnography of Wikipedia* (Stanford University Press).

References

1. Giles J. Internet encyclopaedias go head to head. Nature. 2005;438:900–1.

2. Jemielniak D, Wilamowski M. Cultural diversity of quality of information on Wikipedias. Journal of the Association for Information Science and Technology. 2017;68:2460–70.

3. Michelucci P, Dickinson JL. The power of crowds. Science. 2016;351:32–3.

4. James R. WikiProject Medicine: Creating Credibility in Consumer Health. J Hosp Librariansh. 2016;16:344–51.

5. Mesgari M, Okoli C, Mehdi M, Nielsen FÅ, Lanamäki A. “The sum of all human knowledge”: A systematic review of scholarly research on the content of Wikipedia. Journal of the Association for Information Science and Technology. 2015;66:219–45.

6. London DA, Andelman SM, Christiano AV, Kim JH, Hausman MR, Kim JM. Is Wikipedia a complete and accurate source for musculoskeletal anatomy? Surg Radiol Anat [Internet]. 2019; Available from: http://dx.doi.org/10.1007/s00276-019-02280-1

7. Jemielniak D, Aibar E. Bridging the gap between Wikipedia and academia. Journal of the Association for Information Science and Technology. 2016;67:1773–6.

8. Shafee T, Mietchen D, Su AI. Academics can help shape Wikipedia. Science. 2017;357:557–8.

9. Mietchen D, Wodak S, Wasik S, Szostak N, Dessimoz C. Submit a Topic Page to PLOS Computational Biology and Wikipedia. PLoS Comput Biol. 2018;14:e1006137.

10. Tsueng G, Good BM, Ping P, Golemis E, Hanukoglu I, van Wijnen AJ, et al. Gene Wiki Reviews-Raising the quality and accessibility of information about the human genome. Gene. 2016;592:235–8.

Reply to the reviewer

Click here to access/download
Supplementary Material
gigascience - reply.docx

Click here to access/download
Supplementary Material
GigaScience -Wikipedia_REVISIONS TRACKED.docx
