# Supplementary material for: Wikipedia: Why is the common knowledge resource still neglected by academics?
Source: Gigascience. 2019 Dec 3;8(12):giz139. doi: 10.1093/gigascience/giz139 (PMC6889752; doi:10.1093/gigascience/giz139)
Supplement: giz139_Reviewer_1_Report_Original_Submission — Thomas Shafee -- 10/18/2019 Reviewed [file giz139_reviewer_1_report_original_submission.pdf]

Reviewer Report

Title: Wikipedia: why is the common knowledge resource still neglected by academics?

Version: Original Submission **Date:** 10/18/2019

Reviewer name: Thomas Shafee

Reviewer Comments to Author:

This is a clearly written and timely commentary. It posits that academic engagement in Wikipedia is below where it should be, and speculates on the main reasons why (with the author's opinions on appropriate rebuttals). The author has been appropriate in using recent references (since Wikipedia changes relatively rapidly) and older references are interpreted in context.
My only major comment is that the issue is still presented as static. Has Wikipedia's quality clanged over time? Have the reasons for its neglect also changed, or remained static? It may be worth separating in the "historic bias" paragraph which points are relevant to bias from long-term historical perceptions on what constitutes authority, or from shorter-term perceptions based on Wikipedia's early reputation.
Background
==========
P2: It may be worth noting that there is considerable variation in quality between articles within languages.
P2: "even in very specialized topics" should eb qualified as either in "many specialized topics" or "often in". there have been sources that have found against Wikipedia's quality in some cases. It is also worth noting that the references supporting accuracy in specialised topics often focus on more highly-trafficked pages. Nevertheless, the majority of Wikipedia articles remain 'stub' or 'start' quality level.
Main text
=========
P1: The authors make a good point that the 'anyone can edit' nature can cause suspicion in academics. Is there any known correlation between more-edited articles and accuracy?
P1: The argument that vandalism that is not immediately picked up by bots is mainly obvious and doesn't misinform readers is a risky. I think it would be more appropriate to reverse the sentence and note the machine learning and human editor patrols first (and hence the fast reversion rates), then concede that the sorts of vandalism that passes through may misinform readers but is orders of magniturde rarer.
P3: It may be worth noting that the normal reliance on author credentials/reputation for authority is compensated for by the deliberate mechanism of heavy emphasis on reliable sources and verifiability as Wikipedia's foundation of authority.
P3: It may also be worth noting that despite the 'anyone can edit' fear, more-edited articles are actually tyically more accurate (Per Wilkinson and Huberman 2007. "Cooperation and quality in wikipedia").
P4: May be worth emphasising academic honest and transparency. Perhaps the taboo should be more accurately be against citing _only_ wikipedia.
P6: Not only "paragon of scholarly effort" but transferable information literacy skills.

Conclusions
===========
The conclusions are a fair summary of the main points of the commentary.

Level of Interest

Please indicate how interesting you found the manuscript: Choose an item.

Quality of Written English

Please indicate the quality of language in the manuscript: Choose an item.

Declaration of Competing Interests

Please complete a declaration of competing interests, considering the following questions:

- Have you in the past five years received reimbursements, fees, funding, or salary from an organisation that may in any way gain or lose financially from the publication of this manuscript, either now or in the future?
- Do you hold any stocks or shares in an organisation that may in any way gain or lose financially from the publication of this manuscript, either now or in the future?
- Do you hold or are you currently applying for any patents relating to the content of the manuscript?
- Have you received reimbursements, fees, funding, or salary from an organization that holds or has applied for patents relating to the content of the manuscript?
- Do you have any other financial competing interests?
- Do you have any non-financial competing interests in relation to this paper?

If you can answer no to all of the above, write 'I declare that I have no competing interests' below. If your reply is yes to any, please give details below.

I declare that I have no competing interests

I agree to the open peer review policy of the journal. I understand that my name will be included on my report to the authors and, if the manuscript is accepted for publication, my named report including any attachments I upload will be posted on the website along with the authors' responses. I agree for my report to be made available under an Open Access Creative Commons CC-BY license (http://creativecommons.org/licenses/by/4.0/). I understand that any comments which I do not wish to be included in my named report can be included as confidential comments to the editors, which will not be published.

Choose an item.

To further support our reviewers, we have joined with Publons, where you can gain additional credit to further highlight your hard work (see: https://publons.com/journal/530/gigascience). On publication of this paper, your review will be automatically added to Publons, you can then choose whether or not to claim your Publons credit. I understand this statement.

Yes Choose an item.
