# Supplementary material for: Wikipedia: Why is the common knowledge resource still neglected by academics?
Source: Gigascience. 2019 Dec 3;8(12):giz139. doi: 10.1093/gigascience/giz139 (PMC6889752; doi:10.1093/gigascience/giz139)
Supplement: giz139_Supplemental_Files [file giz139_supplemental_files.zip › gigascience - reply.docx]

Dear Dr Nogoy,

I appreciate the Reviewer’s comments and I’m implementing the suggested changes in full. Comments in-line:

Reviewer reports:
Reviewer #1: This is a clearly written and timely commentary. It posits that academic engagement in Wikipedia is below where it should be, and speculates on the main reasons why (with the author's opinions on appropriate rebuttals). The author has been appropriate in using recent references (since Wikipedia changes relatively rapidly) and older references are interpreted in context.

My only major comment is that the issue is still presented as static. Has Wikipedia's quality clanged over time? Have the reasons for its neglect also changed, or remained static? It may be worth separating in the "historic bias" paragraph which points are relevant to bias from long-term historical perceptions on what constitutes authority, or from shorter-term perceptions based on Wikipedia's early reputation.

For space limitations, I’m reluctant to write a whole paragraph, but to address this issue I’m adding a sentence:

Over time Wikipedia’s quality improved significantly, and yet it is still perceived in a static way, as from the times of its inception.

Background
==========

P2: It may be worth noting that there is considerable variation in quality between articles within languages.

Good point. Adding “…, and also between articles within languages” to the sentence:

Admittedly, standards of quality are shaped by peer-to-peer local language communities and vary largely among Wikipedia projects, and also between articles within languages

P2: "even in very specialized topics" should eb qualified as either in "many specialized topics" or "often in". there have been sources that have found against Wikipedia's quality in some cases. It is also worth noting that the references supporting accuracy in specialised topics often focus on more highly-trafficked pages. Nevertheless, the majority of Wikipedia articles remain 'stub' or 'start' quality level.

Agreed, changed to “many specialized topics” per suggestion.

Main text
=========

P1: The authors make a good point that the 'anyone can edit' nature can cause suspicion in academics. Is there any known correlation between more-edited articles and accuracy?

This is an excellent question that does not have, up to my knowledge, a simple answer, as accuracy is measured differently across topics. Quite a few authors claim that editing leads to rapid improvement, and according to Clay Shirky stubs are much more likely to foster article development than red links. Given that we’re at capacity with references, I’m reluctant to discuss this issue in the article, as we do not have solid universal number to support it.

P1: The argument that vandalism that is not immediately picked up by bots is mainly obvious and doesn't misinform readers is a risky. I think it would be more appropriate to reverse the sentence and note the machine learning and human editor patrols first (and hence the fast reversion rates), then concede that the sorts of vandalism that passes through may misinform readers but is orders of magniturde rarer.

Agreed, revised.

P3: It may be worth noting that the normal reliance on author credentials/reputation for authority is compensated for by the deliberate mechanism of heavy emphasis on reliable sources and verifiability as Wikipedia's foundation of authority.

Agreed, added: “In fact, Wikipedia systematically compensates for the lack of credentials by heavy emphasis on reliable sources.”

P3: It may also be worth noting that despite the 'anyone can edit' fear, more-edited articles are actually tyically more accurate (Per Wilkinson and Huberman 2007. "Cooperation and quality in wikipedia").

Agreed, added for clarity to the sentence about Linus’ law (…”, and the more edited articles are actually more accurate”).

P4: May be worth emphasising academic honest and transparency. Perhaps the taboo should be more accurately be against citing _only_ wikipedia.

Agreed, added “Academic honesty and transparency are crucial for scholarly work, and it is difficult to understand why citing specifically Wikipedia is such a taboo.”

P6: Not only "paragon of scholarly effort" but transferable information literacy skills.

Agreed, added.
